# Co-localization of CENP-C and CENP-H to discontinuous domains of CENP-A chromatin at human neocentromeres

**DOI:** 10.1186/gb-2007-8-7-r148

**Published:** 2007-07-25

**Authors:** Alicia Alonso, Björn Fritz, Dan Hasson, György Abrusan, Fanny Cheung, Kinya Yoda, Bernhard Radlwimmer, Andreas G Ladurner, Peter E Warburton

**Affiliations:** 1 Department of Genetics and Genomic Sciences, Mount Sinai School of Medicine, 1425 Madison Avenue, New York, New York 10029, USA; 2 Gene Expression Unit, Meyerhofstrasse, European Molecular Biology Laboratory (EMBL), 69117 Heidelberg, Germany; 3 Abbott Germany, Max-Planck-Ring, 65205 Wiesbaden, Germany; 4 Bioscience and Biotechnology Center, Nagoya University, Furo-cho, Chikusa-ku, Nagoya 464-8601, Japan; 5 Deutsches Krebsforschungszentrum (DKFZ), Im Neuenheimer Feld, 69120 Heidelberg, Germany

## Abstract

**Background:**

Mammalian centromere formation is dependent on chromatin that contains centromere protein (CENP)-A, which is the centromere-specific histone H3 variant. Human neocentromeres have acquired CENP-A chromatin epigenetically in ectopic chromosomal locations on low-copy complex DNA. Neocentromeres permit detailed investigation of centromeric chromatin organization that is not possible in the highly repetitive alpha satellite DNA present at endogenous centromeres.

**Results:**

We have examined the distribution of CENP-A, as well as two additional centromeric chromatin-associated proteins (CENP-C and CENP-H), across neocentromeric DNA using chromatin immunoprecipitation (ChIP) on CHIP assays on custom genomic microarrays at three different resolutions. Analysis of two neocentromeres using a contiguous bacterial artificial chromosome (BAC) microarray spanning bands 13q31.3 to 13q33.1 shows that both CENP-C and CENP-H co-localize to the CENP-A chromatin domain. Using a higher resolution polymerase chain reaction (PCR)-amplicon microarray spanning the neocentromere, we find that the CENP-A chromatin is discontinuous, consisting of a major domain of about 87.8 kilobases (kb) and a minor domain of about 13.2 kb, separated by an approximately 158 kb region devoid of CENPs. Both CENP-A domains exhibit co-localization of CENP-C and CENP-H, defining a distinct inner kinetochore chromatin structure that is consistent with higher order chromatin looping models at centromeres. The PCR microarray data suggested varying density of CENP-A nucleosomes across the major domain, which was confirmed using a higher resolution oligo-based microarray.

**Conclusion:**

Centromeric chromatin consists of several CENP-A subdomains with highly discontinuous CENP-A chromatin at both the level of individual nucleosomes and at higher order chromatin levels, raising questions regarding the overall structure of centromeric chromatin.

## Abstract 2

The distribution of centromeric chromatin-associated proteins across human neocentromeric DNA shows that this chromatin consists of several CENP-A-containing sub-domains.

## Background

The centromere, which is the chromosome component that is responsible for the proper segregation of sister chromatids to daughter cells during cell division, is a specialized chromatin structure [1,2]. Centromeric chromatin has a distinctive nucleosome structure that contains the histone H3 variant centromere protein (CENP)-A [3-8]. CENP-A containing chromatin associates with a large number of proteins, which are assembled in a hierarchical manner [9-12]. Essential among the proximal proteins are several associated with the centromere throughout the cell cycle, such as CENP-C (a DNA-binding protein) [13-18] and CENP-H (necessary for CENP-C loading) [16,19,20]. These proteins provide the platform onto which the mitotic kinetochore is assembled, with CENP-A potentially providing the epigenetic mark that specifies centromere formation [21,22].

Immunofluorescence studies of extended chromatin fibers at human endogenous centromeres have demonstrated that human centromeres are formed by discontinuous CENP-A nucleosome domains of about 15 to 40 kilobases (kb), interspersed with nucleosome domains containing modified histone H3 dimethylated at Lys4 [23,24]. These domains form on arrays of 0.5 to 1.5 megabases (Mb) of a family of tandemly repeated DNA called alpha satellite [25], binding primarily to the alpha I subset of these sequences [26,27]. In metaphase chromosomes it has been postulated that the histone H3 domains face inward toward regions of sister chromatid cohesion, whereas the CENP-A domains face poleward and assemble the kinetochore [23].

Human neocentromeres are variant centromeres that have arisen epigenetically on low-copy complex genomic DNA. Over 75 cases have been reported on derivatives of at least 19 different human chromosomes, identified mainly through clinical chromosomal analysis [28]. They assemble fully functional kinetochores with the sole absence of CENP-B, which is known to bind alpha satellite DNA [29]. Thus, they have been used as a model system in which to study the underlying centromeric chromatin in the absence of repetitive alpha satellite DNA. Using chromatin immunoprecipitation (ChIP) and bacterial artificial chromosome (BAC) microarrays, the CENP-A chromatin domain of six different neocentromeres has been described. These range from 130 kb to 460 kb in size, which is about twofold to threefold smaller than alpha satellite DNA arrays found at endogenous centromeres [30-33]. In addition, the CENP-C chromatin domain was described on a seventh neocentromere to an approximately 54 kb domain [33]. ChIP and BAC microarray analysis of multiple independent neocentromeres that formed in so-called neocentromere 'hotspots' [28,29], specifically three from band 13q32 [32] and two from band 13q21 [33], show that they formed in distinct genomic regions separated by up to several megabases, suggesting little role for primary DNA sequence determinants in neocentromere formation. Further analysis of a neocentromere in band 10q25 (the mardel10 chromosome) using a polymerase chain reaction (PCR) amplicon microarray (with an average fragment size of 8 kb) has demonstrated that CENP-A nucleosomes at this neocentromere are organized into seven distinct CENP-A subdomains [34].

In this study we have analyzed the binding sites for CENP-A, CENP-C, and CENP-H in human neocentromeres from band 13q32, using BAC, PCR-amplicon, and oligonucleotide-type genomic microarrays. BAC microarray analysis of two neocentromeres showed that both CENP-C and CENP-H co-localized to the same chromatin domain as CENP-A. The high-resolution PCR-amplicon microarray analysis reported here showed that a 130 kb CENP-A domain previously indicated by low-resolution BAC microarray analysis [32] actually consisted of an approximately 87.8 kb major domain and an approximately 13.2 kb minor domain, separated by about 158 kb. Both domains contained co-localizing CENP-A, CENP-C, and CENP-H, indicating a distinct inner kinetochore chromatin structure. Analysis of CENP-A ChIP DNA hybridized to a high-resolution 70 mer oligonucleotide microarray containing two regions within the major domain showed that the density of CENP-A nucleosomes may be highly variable in some regions within the neocentromeric domains.

## Results

### Co-localization of CENP-A, CENP-C, and CENP-H positions on two independent 13q32/33 neocentromeres

**Figure 1:**
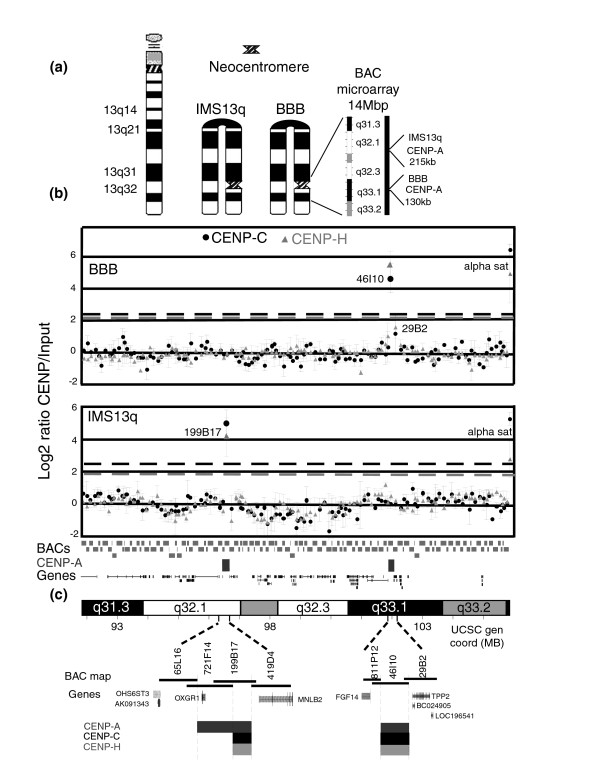
Genomic microarray analysis of CENP-C and CENP-H binding domains in two independent 13q32/33 neocentromeres. **(a) **Ideogrammatic representation of the two neocentric chromosomes analyzed. From left to right: a normal chromosome 13, the invdup13q21 in IMS13q with a neocentromere in band 13q32, and the invdup13q21 in BBB with a neocentromere in band 13q33.1. An expansion of the 13q31.3 to 13q33.2 area included in the bacterial artificial chromosome (BAC) CHIP is shown. The position and size of each previously mapped centromere protein (CENP)-A domain from Alonso and coworkers [32] are indicated. **(b) **DNA obtained from chromatin immunoprecipitation (ChIP) using antibodies to CENP-C (circles) and CENP-H (triangles) from cell lines BBB and IMS13q was hybridized to a contiguous BAC microarray spanning 14 megabases (Mb) from 13q31.3 to 13q33.2. Shown across the bottom of the graph is the tiling path of the unique sequenced regions for each BAC, the previously determined CENP-A domains [32] in cell lines BBB and IMS13q, and the genes in the region. Three independent biologic replicates were performed for each ChIP from each cell line, and the scale normalized mean log_2 _Cy-5:Cy-3 intensity ratios (ChIP to input) with standard error (SE) were plotted on the y-axis for each BAC. Positive intensity ratios were identified as those that were at least three times the standard deviation (SD) from the experimental mean (gray or black dashed lines; see Materials and methods). For cell line BBB, CENP-C ChIP, the experimental mean was 0 ± 0.82 SD. Positive values ≥ 2.5 (black dashed line) were as follows: alpha sat = 6.42 ± 0.39 SE and BAC RP11-46I10 = 4.66 ± 0.92 SE. BAC RP11-29B2 was slightly increased (1.18 ± 1.2 SE) but not statistically significantly. All other BACs ranged from -1.1 to ≤ 0.96. For cell line BBB, CENP-H ChIP, the experimental mean was -0.02 ± 0.75 SD. Positive values ≥ 2.2 (grey dashed line) were as follows: alpha sat = 4.92 ± 1.86 SE and BAC RP11-46I10 = 5.57 ± 0.77 SE. BAC RP11-29B2 was slightly increased (1.58 ± 0.71 SE) but not statistically significantly. All other BACs ranged from -1.27 to ≤ 1.03. For cell line IMS13q, CENP-C ChIP, the experimental mean was 0 ± 0.84 SD. Positive values ≥ 2.5 (black dashed line) were as follows: alpha sat = 5.26 ± 0.38 SE and BAC RP11-199B17 = 4.95 ± 0.86 SE. All other BACs ranged from -1.7 to ≤ 0.93. For cell line IMS13q, CENP-H, the experimental mean was 0.00 ± 0.64 SD. Positive values ≥ 1.9 (grey dashed line) were as follows: alpha sat = 2.63 ± 1.03 SE and BAC RP11-199B17 = 3.95 ± 1.06 SE. All other BACs ranged from -1.17 to ≤ 1.13. **(c) **Expansion of BAC map in regions that are positive for CENP-C and CENP-H in each neocentromere examined, showing BAC names and overlaps, the genes, and the previously determined CENP-A domains. For cell line BBB, the CENP-A, CENP-C, and CENP-H were mapped to the identical BACs (negative for RP11-811P12, strongly positive for BAC 46I10, and weakly positive for 29B2). For cell line IMS13q, the CENP-A mapped to two contiguous BACs (RP11-721F4 and RP11-199B17), whereas CENP-C and CENP-H mapped only to one BAC (RP11-199B17).

We sought to determine the DNA localization of the two constitutive centromere proteins CENP-C and CENP-H relative to CENP-A domains at neocentromeres. Cell line BBB contains an inverted duplication chromosome with a breakpoint in band 13q21 (invdup13q21) and a neocentromere in band 13q33.1 (Figure 1a) [32,35]. The CENP-A domain was previously localized using CENP-A ChIP to a 130 kb domain encompassing the unique sequences in BAC RP11-46I10 and the overlapping sequences with the contiguous BAC RP11-29B2 [32]. To identify the CENP-C domain in this cell line, CENP-C ChIP was performed on formaldehyde cross-linked BBB extracts (see Materials and methods, below) and the DNA hybridized to contiguous BAC microarrays spanning chromosome bands 13q32 and 13q33.2 (Figure 1). BAC RP11-46I10 showed a highly significant positive signal and BAC RP11-29B2 was slightly positive, but not above the cut-off for statistical significance (dashed line, Figure 1b). This pattern was similar to the pattern previously seen for CENP-A ChIP in this cell line [32]. Alpha satellite DNA, included on the BAC arrays as a positive ChIP control at endogenous centromeres, showed a highly significant positive signal.

CENP-H ChIP was also performed on formaldehyde cross-linked BBB extracts, and the DNA hybridized to the BAC microarrays. Again, BAC RP11-46I10 gave an enhanced signal, and BAC RP11-29B2 gave a positive but weaker signal that was not above the cut-off for statistical significance (dashed line, Figure 1b). The alpha satellite control also gave a highly significant positive signal. The exact correlation of BAC hybridization for CENP-A [32], CENP-C, and CENP-H indicate the precise co-localization of these three inner kinetochore proteins at this neocentromere at the resolution of this microarray. As a negative control, both CENP-C and CENP-H ChIPs derived from either HeLa (no neocentromere) or CHOP13q (neocentromere in 13q21) [33] showed no positive hybridization signal in this microarray except for the alpha satellite positive control (data not shown).

To assess this co-localization in an independent neocentromere cell line, CENP-C and CENP-H ChIP were performed on cell line IMS13q, which has an invdup 13q21 chromosome with a neocentromere in band 13q32.1 (Figure 1a) [32,35]. The CENP-A domain in this neocentromere was previously localized using CENP-A ChIP to a 215 kb domain approximately 5 Mb proximal to that of BBB (Figure 1a), based on the positive signal at two contiguous BACs, RP11-721F14 and RP11-199B17 [32]. For both the CENP-C and CENP-H ChIP, BAC RP11-199B17, but not RP11-721F4, was positive (the alpha satellite controls were also positive; Figure 1b). These data indicate that CENP-A, CENP-C, and CENP-H also co-localize at this neocentromere. However, the lack of detectable hybridization of CENP-C and CENP-H at BAC RP11-721F14 suggest an approximately 70 kb domain where CENP-A, CENP-C, and CENP-H co-localize, with the CENP-A domain extending an additional 146 kb (based on the BAC overlaps; Figure 1c). However, given the lower efficiency of immunoprecipitation of CENP-C and CENP-H associated DNA compared with CENP-A, we cannot determine whether smaller domains of CENP-C and CENP-H are also found on BAC RP11-721F14 that correspond to the CENP-A signal (see below). These data show that the both CENP-C and CENP-H proteins co-localize with the histone variant CENP-A chromatin domains at two independent human neocentromeres.

### CENP-A chromatin in the BBB neocentromere is organized into two distinct domains

**Figure 2:**
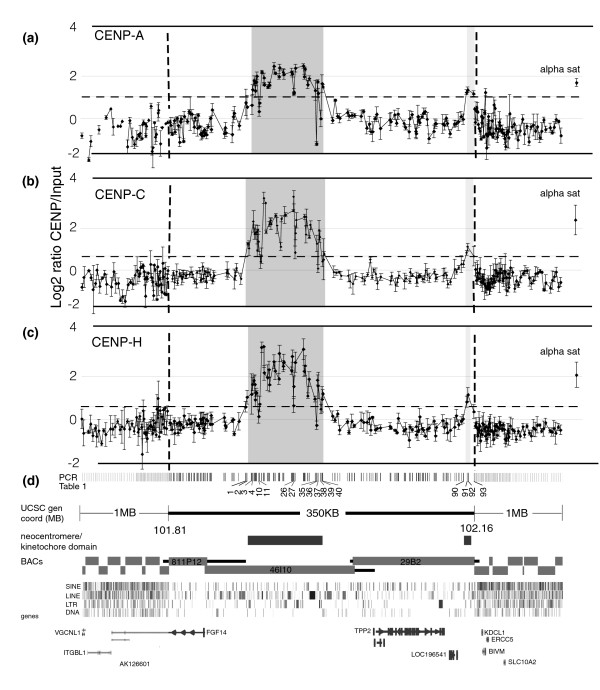
The BBB neocentromere contains a major and a minor centromere chromatin domain. DNA obtained from chromatin immunoprecipitation (ChIP) using antibodies to CENP-A, CENP-C, and CENP-H from cell line BBB was hybridized to a custom made microarray containing 257 unique polymerase chain reaction (PCR) fragments. Three independent biological replicates were performed for each antibody, and the scale normalized mean log_2 _Cy-5:Cy-3 intensity ratios (ChIP to input), were plotted on the y-axis with the standard error (SE) for each PCR fragment. Intensity ratios at least three times the standard deviation (SD) from the background mean (dashed line) were considered positives (see Materials and methods). An alpha satellite containing plasmid was included as a positive control (far right). **(a) **Centromere protein (CENP)-A ChIP. The major CENP-A domain was about 80.3 kilobases (kb; shaded region), with positive intensity ratios 1.17 to 2.46. The minor domain was about 8.5 kb (shaded region) and was approximately 162 kb downstream from the major domain; intensity ratios were 1.14 to 1.33. Background experimental mean was -0.39 ± 0.47 SD, one-tailed distribution cut-off was ≤ 0.68, positive values were ≥ 1.02 (dashed line). Alpha satellite = 1.63 ± 0.18 SE. **(b) **CENP-C ChIP. Major CENP-C domain was 87.8 kb (shaded region). Intensity ratios were 0.67 to 3.41. Minor domain was 8.5 kb; intensity ratios were 0.65 to 1.07 (shaded region). Background experimental mean was -0.37 ± 0.34 SD, one-tailed distribution cut-off was ≤ 0.31, positive values were ≥ 0.65 (dashed line). Alpha satellite = 2.36 ± 0.70 SE. **(c) **CENP-H ChIP. Major CENP-H domain was about 86.3 kb (shaded region), and positive intensity ratios were 0.64 to 3.35. Minor domain was about 1.9 kb (shaded region), and intensity ratios were 0.82 and 1.14. Background experimental mean was -0.33 ± 0.32 SD, one-tailed distribution cutoff was ≤ 0.56, positive values were ≥ 0.63 (dashed lines). Alpha sat = 2.06 ± 0.59 SE. **(d) **The 2.3 megabase (Mb) region included in the PCR CHIP. The central 350 kb region, covered by PCR fragments at high density. The adjacent megabase on either side of the central region, shown at a 10 fold reduced scale, was covered by PCR fragments at decreasing density. PCR microarray fragments listed in Table 1, found at the edges of CENP-A, CENP-C and CENP-H domains, and the negative values within the first domain, are shown. The major and minor chromatin domains are shown by the rectangles. The tiling path of the unique sequenced regions of each bacterial artificial chromosome (BAC) and their overlaps are shown within the 350 kb region. The corresponding Repeat Masker data from the Human Genome Browser at UCSC and thegenes in the area are indicated [50].

In order to further analyze the organization of the 130 kb CENP-A chromatin domain at the BBB neocentromere, a microarray that increases the resolution of this region by about 200-fold was constructed. This microarray contains 257 PCR amplified fragments that span 2.3 Mb of genomic DNA surrounding and encompassing the 130 kb CENP-A domain predicted by the BAC CHIP. Primer pairs were designed in the unique DNA sequences located between the interspersed repetitive DNA in this region. PCR products ranged in size from 173 base pairs (bp) to 942 bp in length, with a mean value of 588 bp. A set of 133 PCR products were designed to saturate the 350 kb region that includes the sequences in BACs RP11-811P12, RP11-46I10, and RP11-29B2, with an average spacing of about 2 kb (Figure 2d). The remaining 124 primer pairs were designed to cover about 1 Mb on either side of the 350 kb region, positioned at regular intervals with increasing spacing from 10 kb to 30 kb as they moved away from the central 350 kb region (Figure 2d; also see Material and methods, below). Also included as a positive control is a plasmid containing 500 bp of the alpha satellite DNA sequence found at the centromeric regions of chromosomes 1, 5, and 9.

Hybridization of this PCR microarray with CENP-A ChIP DNA showed two regions of CENP-A containing chromatin within the central 350 kb. Positive intensity ratios were determined using a one-tailed distribution analysis (dashed line, Figure 2a; see Materials and methods, below) [36]. These CENP-A domains did not coincide with the original prediction of 130 kb domain based on the BACs overlaps (Figure 1) [32]. Instead, the PCR microarray indicated two distinct domains of CENP-A chromatin: a major domain of about 80.3 kb in size and a minor domain of about 8.5 kb (Figure 2a). The major domain was located within the unique sequence of BAC RP11-46I10 and accounted for the strong hybridization signal to this BAC on BAC microarrays [32]. The minor domain was delineated by three neighboring PCR fragments, 91, 92 and 93 (Figure 2d and Table 1). It is approximately 162 kb downstream of the major domain at the distal end of BAC RP11-29B2 and not within the region that overlaps with BAC RP11-46I10. This minor domain accounts for the weak but consistent hybridization signal seen for BAC RP11-29B2 on BAC microarrays [32]. As a negative control, CENP-A ChIP derived from cell line CHOP13q [33] showed no positive hybridization signal in this microarray except for the alpha satellite positive control (data not shown).

**Table 1:** PCR microarray and qRT-PCR values across the CENP-A major and minor domains

**PCR fragment name^a^**	**Distance from fragment 1^b^**	**Size (bp)**	**qRT-PCR value (Figure 3)**	**CENP-A ChIP cut-off ≤ 0.68, positive ≥ 1.02^c^**	**CENP-C ChIP cut-off ≤ 0.31, positive ≥ 0.65^c^**	**CENP-H ChIP cut-off ≤ 0.56, positive ≥ 0.63^c^**	**UCSC Human Genome Browser (hg17) genome coordinates**
**1**	**0**	**569**		**0.72(-/+)**	**1.23(+)**	**0.59(-/+)**	**101,899,991-101,900,560**
**(1)**	**316**	**247**	**1.01(+)**				**101,900,060-101,900,307**
**2**	**2,064**	**566**		**0.51(-)**	**1.03(+)**	**1.03(+)**	**101,901,489-101,902,055**
**(2)**	**1,965**	**150**	**1.15(+)**				**101,901,806-101,901,956**
**C**	**6,166**	**178**	**1.78(+)**				**101,905,979-101,906,157**
**3**	**7,462**	**796**		**0.60(-)**	**2.28(+)**	**1.17(+)**	**101,906,657-101,907,453**
**(3)**	**7,184**	**213**	**2.22(+)**				**101,906,962-101,907,175**
**4**	**8,225**	**706**		**1.34(+)**	**1.70(+)**	**1.62(+)**	**101,907,510-101,908,216**
D	12,840	223	1.80(+)				101,912,608-101,912,831
10	15,300	423		0.31(-)	0.40(-)	0.15(-)	101,914,868-101,915,291
11	16,222	649		0.71(-/+)	0.67(+)	0.72(+)	101,915,564-101,916,213
(11)	15,830	247	2.76(+)				101,915,574-101,915,821
**26**	**53,983**	**861**		**1.17(+)**	**0.83(+)**	**0.64(+)**	**101,953,974-101,954,835**
**27**	**55,119**	**610**		**1.21(+)**	**1.74(+)**	**1.53(+)**	**101,954,501-101,955,110**
**(27)**	**54,554**	**190**	**3.44(+)**				**101,954,355-101,954,545**
35	80,686	649		-1.21(-)	0.07(-)	-0.25(-)	101,980,028-101,980,677
(35-1)	80,226	172	0.86(-)				101,980,045-101,980,217
(35-2)	80,432	203	0.73(-)				101,980,220-101,980,423
36	81,822	450		1.72(+)	1.88(+)	1.82(+)	101,981,363-101,981,813
37	82,493	350		1.17(+)	1.56(+)	1.56(+)	101,982,134-101,982,484
38	85,610	786		-0.03(-)	0.41(-/+)	0.50(-)	101,984,815-101,985,601
(38)	85,501	143	0.48(-)				101,985,349-101,985,492
39	87,099	924		1.62(+)	0.50(-/+)	0.93(+)	101,986,166-101,987,090
40	87,859	779		1.49(+)	0.76(+)	1.19(+)	101,987,071-101,987,850
**90**	**246,368**	**430**		**-0.06(-)**	**0.29(-)**	**0.04(-)**	**102,145,930-102,146,359**
**(90)**	**246,170**	**231**	**1.12(+)**				**102,145,930-102,146,161**
**91**	**250,586**	**597**		**1.21(+)**	**0.80(+)**	**0.82(+)**	**102,149,980-102,150,577**
**(91)**	**250,432**	**187**	**1.43(+)**				**102,150,236-102,150,423**
**92**	**251,863**	**534**		**1.33(+)**	**1.07(+)**	**1.14(+)**	**102,151,320-102,151,854**
**(92)**	**251,830**	**236**	**1.34(+)**				**102,151,585-102,151,821**
**S**	**253,879**	**158**	**2.65(+)**				**102,153,712-102,153,870**
**T**	**255,702**	**189**	**4.22(+)**				**102,155,504-102,155,693**
**93**	**258,459**	**481**		**1.14(+)**	**0.65(+)**	**0.37(-)**	**102,157,969-102,158,450**
**(93)**	**258,331**	**245**	**2.30(+)**				**102,158,077-102,158,322**
**U**	**259,157**	**224**	**0.99(+)**				**102,158,924-102,159,148**
**V**	**265,411**	**169**	**0.26(-)**				**102,165,233-102,165,402**

^a^Numbers indicate polymerase chain reaction (PCR) microarray fragments as in Figure 2; numbers in parentheses indicate quantitative real-time (qRT)-PCR fragments wholly contained within the PCR microarray fragment with same number (Figure 3); letters indicate qRT-PCR fragments only (Figure 3). Different regions within the major domain and the minor domain are indicated by alternating bold and regular rows. ^b^Distances are taken from the first coordinate in fragment 1 to the last coordinate of the next fragment. ^c^Value given in Figure 2 for PCR microarray chromatin immunoprecipitation (ChIP): (+), ≥ threshold for positive values; (±), weakly positive, over cut-off but below the threshold for positive values; (-), below the cut-off value. CENP, centromere protein.

**Figure 3:**
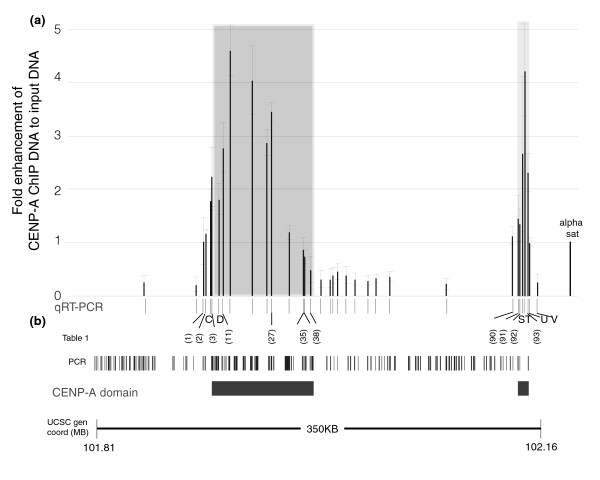
qRT-PCR confirms two separate CenpA domains in the neocentromeric cell line BBB. **(a) **Quantitative real-time polymerase chain reaction (qRT-PCR) was performed on equal amounts of total DNA obtained from centromere protein (CENP)-A chromatin immunoprecipitation (ChIP) DNA and Input DNA from BBB cell line. The thirty-four PCR primer pairs used (shown as black lines in the x-axis) amplified fragments from 150 to 250 base pairs contained within the 350 kb neocentromere region (see Figure 2). Each primer pair was assayed in at least three independent CENP-A ChIP experiments. The qRT-PCR results for each primer pair were expressed on the y-axis as the fold enhancement between the CENP-A ChIP DNA and input DNA (= 1.93^ΔCt(CENP-A-Input)^) normalized to the value obtained for the positive control alpha satellite DNA primer pair (far right). The shaded region indicates the area determined to be the CENP-A domain in Figure 2. **(b) **The 34 qRT-PCR primer pairs and the 133 PCR products from this region on the PCR microarray (Figure 2) are shown. qRT-PCR primers that amplified products wholly contained within a PCR microarray fragment are indicated by numbers in parentheses; the rest are labeled alphabetically. Only qRT-PCR fragments shown in Table 1 are indicated; information for all other primers can be found in the Additional data file 3. CENP-A domains derived from the PCR microarray data are indicated. Genome coordinates correspond to the region of chr13 from the Human Genome Browser at UCSC (hg17) [50].

In order to validate the PCR microarray data, we assayed CENP-A enrichment over the neocentromeric chromatin regions using quantitative real-time (qRT)-PCR analysis of CENP-A ChIP DNA compared with input DNA (Figure 3a). Thirty-four PCR primer pairs were used to amplify fragments of about 200 bp across the 350 kb region, which confirmed the presence of both the major and minor CENP-A domains and intervening regions, and validated the PCR microarray results (Figure 3 and Table 1). Some differences were observed between the PCR microarray and the qRT-PCR analysis, especially from fragments near the edges of the domains and fragments that showed relatively low intensity ratios within the major domain. The three PCR microarray fragments 1, 2, and 3 on the most 5' edge of the major domain were below the threshold for positive values on the PCR microarray, although they had relatively high values compared with the intervening regions (Figure 2a and Table 1). qRT-PCR analysis using primers that amplified fragments contained within these larger PCR microarray fragments gave low but increasingly positive values (Figure 3 and Table 1), suggesting the presence of CENP-A and extending the 5' side of the major domain to include fragments 1, 2, and 3.

Two regions within the major domain represented by fragments 10 and 11, and by fragments 26 and 27 showed negative or strongly reduced signals on the PCR microarray (Figure 2a and Table 1). However, qRT-PCR of DNA contained within fragment 11 and within fragment 27 were both strongly positive for CENP-A (Figure 3 and Table 1), suggesting that these regions were not completely devoid of CENP-A. On the 3' edge of the major domain, PCR microarray fragment 35 (Figure 2a and Table 1) was strongly negative for CENP-A. qRT-PCR of two different fragments from within fragment 35 were both negative (below the signal obtained for the alpha satellite DNA control), although they were significantly higher than the qRT-PCR fragments within the intervening regions (Figure 3 and Table 1), suggesting a small amount of CENP-A at this region. PCR microarray fragment 38 was negative (Figure 2a), which was confirmed by qRT-PCR (Figure 3 and Table 1). PCR microarray fragments 39 and 40 were both weakly positive for CENP-A, defining the 3' edge of the major domain. These data gave a total estimate of 87.8 kb for the major CENP-A domain, encompassing PCR fragments 1 to 40 (Figure 2a,d).

The three PCR microarray fragments 91, 92, and 93 make up the minor domain (Figure 2a), which was confirmed by qRT-PCR (Figure 3 and Table 1). Fragment 90 was negative for CENP-A on the PCR microarray but weakly positive by qRT-PCR (Figure 3 and Table 1). One additional qRT-PCR fragment 3' of 93 was weakly positive, marking the 3' boundary of the minor domain (Figure 3 and Table 1) and giving a size estimate of 13.2 kb. These combined PCR microarray and qRT-PCR data suggest that the CENP-A chromatin is not homogeneous across the major and minor domain, but instead may contain distinct subdomains of differing CENP-A nucleosome density (see below).

### CENP-C and CENP-H domains colocalize with the two discontinuous CENP-A chromatin domains

The low-resolution BAC CHIP analysis (Figure 1) suggested a precise co-localization of CENP-C and CENP-H with CENP-A chromatin at the neocentromere in cell line BBB. The extent of this co-localization was further examined relative to the two CENP-A domains revealed by the PCR microarray and qRT-PCR data described above. Hybridization of both CENP-C and CENP-H ChIP DNA to the PCR microarray showed two domains that closely corresponded to the CENP-A domains. The major CENP-C domain had a size of about 87.8 kb, which included the three 5' most PCR fragments 1, 2, and 3 (Figure 2b and Table 1). The second minor CENP-C domain coincided precisely with the minor CENP-A domain on PCR fragments 91, 92, and 93 (Figure 2b and Table 1). The major CENP-H domain was about 86.4 kb in size, and included the two 5' PCR fragments 2 and 3 (Figure 2c and Table 1). The minor domain was seen to be about 1.9 kb, because only fragments 91 and 92 were significantly positive. Fragments 1 and 93 had relatively high values compared with the intervening regions (Figure 2c and Table 1), suggesting that failure to detect CENP-H in these areas of lower density is most likely due to lack of sensitivity of the CENP-H ChIP. For both CENP-C and CENP-H ChIP, the alpha satellite plasmid showed a highly positive signal intensity ratio. As a negative control, both CENP-C and CENP-H ChIPs derived from cell line CHOP13q (neocentromere in band 13q21 [33]) showed no positive hybridization signal in this microarray except for the alpha satellite positive control (data not shown).

Interestingly, the regions that showed reduced intensity values for the CENP-A domains were largely consistent for both CENP-C and CENP-H, showing definite reduced intensity at both fragments 10 and 11, and at fragments 26 and 27 (Figure 2b,c and Table 1). On the 3' edge of the major domain, PCR microarray fragment 35 (Figure 2 and Table 1) was consistently negative for all three CENPs. PCR microarray fragments 36 and 37 were positive for all three CENPs. Fragment 38 was negative for all three CENPs (Table 1 and Figure 3). Fragment 39 was positive for CENP-A and CENP-H, and weak for CENP-C. Fragment 40 was the most 3' fragment to be positive for all three CENPs, which defined the 3' edge of the major domain (Figure 2 and Table 1).

**Figure 4:**
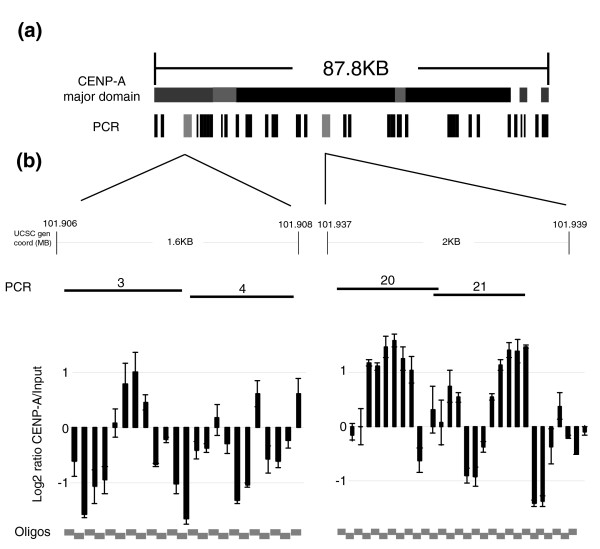
CENP-A nucleosomes are interspersed at variable densities throughout the core centromeric domain. **(a) **The 87.8 kilobase (kb) major domain. Shown are the putative subdomains of centromere protein (CENP)-A, with higher densities indicated by darker shading. The polymerase chain reaction (PCR) microarray fragments are shown below (see Figure 2). The fragments examined using the oligo array are shown in gray. **(b) **DNA obtained from chromatin immunoprecipitation (ChIP) using CENP-A from cell line BBB was hybridized to a 70 mer oligonucleotide microarray containing two distinct subdomains of the major neocentromere domain, a 1.6 kb region at the 5' end (PCR fragments 3 and 4; Table 1) and a 2 kb region within the domain (PCR fragments 20 and 21). Three independent biological replicates were performed, and the mean log_2 _Cy-5:Cy-3 intensity ratio (CENP-A ChIP to input) from each biologic replicate was scale normalized (SN). The result for each 70 mer oligomer is shown plotted on the y-axis with the standard error.

Thus, these results suggest tight co-localization of CENP-A, CENP-C, and CENP-H across the major and minor domains, and define a distinct chromatin structure that contains these three centromeric proteins. Overall, for both the major and minor domains, the PCR microarray intensity ratios suggested the highest density of this CENP chromatin in the interior of the domains and reduced density toward the edges (Figure 2a,b,c). The regions within the major domain with reduced intensity for all three proteins may further define several subdomains within the major domain: a 5' domain of about 13 kb (fragments 1 to 9; Figure 2) with low CENP chromatin density; two central domains of about 35.3 kb (fragments 12 to 25) and 23.4 kb (fragments 28 to 34) with relatively high density; and two small 3' domains with relatively low density of 1.1 kb (fragments 36 and 37) and 1.7 kb (fragments 39 and 40; Figure 4a). Notably, the regions separating these subdomains were no greater than 5 to 6 kb in size, and the PCR microaray and qRT-PCR results were not consistent with complete absence of CENPs but rather with greatly reduced density in these regions.

### CENP-A nucleosomes are interspersed along the major neocentromeric domain

In order to confirm the PCR microarray data that CENP chromatin was not evenly distributed across the major domain, we set out to examine directly the density of CENP-A nucleosomes at the edges and the middle of the major domain. Therefore, we constructed a custom oligo-CHIP that contained a 1.6 kb region on the 5' edge of the domain (fragments 3 and 4; Table 1) and a 2 kb region from within the domain (fragments 20 and 21). Contiguous 70 mers that fully covered these regions were spotted onto a glass slide and hybridized with CENP-A ChIP DNA that was enhanced for mononucleosome size DNA (Figure 4). In the first region (left side, Figure 4b), seven out of 23 oligos (30%) showed a positive intensity ratio, four of which were found in a contiguous stretch, and three of which were noncontiguous. By contrast, in the second region (right side, Figure 4b) 16 out of 28 oligos (57%) showed a positive intensity ratio, with three distinct regions of contiguous oligos and a single noncontiguous one. These results are consistent with the intensity values obtained with the PCR array, and they support low CENP-A occupancy in the first region and high CENP-A occupancy in the second region. The high resolution of these limited oligo data strongly support the suggestion of differing densities of CENP-A nucleosomes in the neocentromere, with a sparse distribution at the beginning of the major domain and a more dense distribution toward the middle of the domain. This heterogeneity in CENP-A density across the neocentromere is unexpected and has important implications for models of centromeric chromatin structure and formation.

### Sequence Analysis of the CENP-A domains

The high-resolution analysis presented here permits further sequence analysis of the major and minor domains and the intervening regions. Analysis of the interspersed repetitive DNA elements showed increases in both long interspersed nucleotide element (LINE)1 and mammalian apparent long terminal repeat retrotransposon (MaLR) within the CENP-A major domain (27% LINE1 and 9.0% MaLR elements) and minor domain (29.3% LINE1 and 4.0% MaLR) as compared with the intervening region (11.8% LINE1 and 2.9% MaLR) and genome average (16.9% LINE1 and 3.65% MaLR). A previously analyzed neocentric marker chromosome mardel10 from 10q25 also showed enrichment for LINE1 elements in CENP-A binding regions relative to intervening regions, although it did not show MaLR enrichment [34]. These two neocentromeres combined with the five other neocentromeres analyzed by BAC ChIP on CHIP provide a large dataset for further analysis of the contribution of DNA sequence, if any, to neocentromere formation.

**Figure 5:**
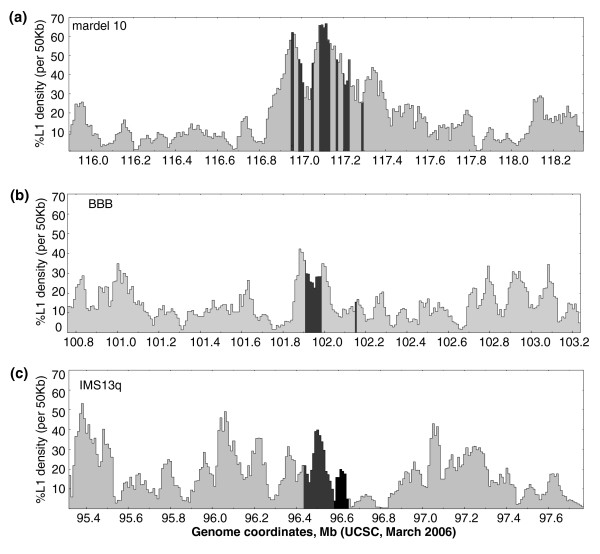
Sliding window analysis of LINE1 density at neocentromeres. The long interspersed nucleotide element (LINE)1 (L1) density of 50 kilobase (kb) windows shifted every 10 kb are shown across 2.5 megabase (Mb) regions centered on human neocentromeres that have been localized by chromatin immunoprecipitation (ChIP) analysis. The hg18 genome coordinates [50] for each region are shown on the x-axis; the density of L1 elements as percentage of base pairs per each 50 kb window are shown on the y axis. **(a) **mardel10, the centromere protein (CENP)-A domains indicated by the shaded regions. The CENP-C domain has not been determined. The CENP-H domain has been mapped to hg17 genome coordinates chr10: 115,058,211 to 115,833,955 [50], which are outside this window [39]. **(b) **BBB. The CENP-A, CENP-C, and CENP-H domains are indicated by the shaded regions. **(c) **IMS13q. Shaded and black region indicate colocalization of CENP-A, CENP-C, and CENP-H, and shaded area indicates extent of CENP-A domain beyond this region. This neocentromere was mapped using lower resolution bacterial artificial chromosome (BAC) microarrays.

Sequence analysis of the density of LINE1 (L1) sequences at these seven neocentromeres was further examined by performing a sliding window analysis of L1 density across 2.5 Mb genomic regions that contain neocentromeres (Figure 5 and Additional data file 1). The 10q25 neocentromere occurred in a region of exceptionally high L1 density, with the CENP-A domains largely centered on the peaks of L1 density, but with many of the individual intervening regions also containing comparably high L1 densities [34], even if overall the average density was significantly lower (Figure 5a). The major domain of the BBB neocentromere is also found at a region of L1 density, although it is flanked by regions of higher density, one of which extends into the intervening sequence (Figure 5b). The neocentromere region in IMS13q also shows the CENP-A domain at a strong local peak of L1 density, although the region that was shown to co-localize with CENP-C and CENP-H falls outside this peak, and there are other regions of comparable L1 density within the 2.5 Mb window analyzed (Figure 1c). The four other neocentromeres that have been localized by CENP-A or CENP-C ChIP [31,33] show varying degrees of L1 density (Additional data file 1). These data taken together suggest a possible role for L1 density in establishing the location of neocentromere formation, especially in regions with large local variation. Whether this is due to some aspect of L1 biology or merely indicates a preference of CENP-A for a DNA motif or profile that occurs more often in L1 sequences remains to be determined.

Sequence analysis of A+T content at neocentromeres was performed by examining first the %AT content of 40 kb windows shifted every 10 kb across 2.5 Mb of the neocentromere domains (Additional data file 2 [part A]). Although the mardel10 neocentromere was highly enriched for AT sequences, no such correlation was observed for any of the 13q neocentromeres examined. Next, 200 bp windows shifted every 50 bp across a 50 Mb region of chromosome 13, which contains five independent 13q neocentromeres, was examined. Many AT-rich clusters were seen; however, these did not correlate with the neocentromere positions (Additional data file 2 [part B]). At the BBB neocentromere there is a peak of AT-rich DNA, but closer analysis shows this DNA is actually found in the intervening region between the major and minor CENP-A domains. Finally, sequences approximating the *Saccharomyces cerevisiae *functional centromeric CDEII sequences (four or more runs of A5-7/T5-7 in 90 bp of ≥ 90% A or T) [37] were located across the 50 Mb region, which did not correlate with the position of neocentromeres (Additional data file 2 [part C]). These analyses do not support a role for AT richness in CENP-A preference for assembly at chromosome 13q neocentromeres.

In order to examine further the possible role of DNA sequence in neocentromere formation, an analysis of oligomers shared between these seven neocentromere positions was performed. The longest motif that is present in at least one copy in all seven neocentromeres is a 70 mer, which is part of most young L1 elements (positions 5576 to 5645 in the L1PA2 consensus sequence, 51% A+T rich; for sequence, see Materials and methods, below). However, this motif occurs 130 times across chromosome 13 with a median distance of about 42 kb, and does not have a higher density in the neocentromere forming regions than elsewhere on chromosome 13. An analysis of all 9 bp oligomers revealed 22,879 different 9 mers present in at least one copy in the neocentromere sites. None of these was found to be present at significantly higher frequency, at each neocentromere, than expected based on their chromosomal distribution [38]. Thus, this sequence analysis of the seven currently described neocentromere regions does not reveal any striking sequence composition that distinguished them from the remainder of the genome, and thus any power to predict additional neocentromere locations is extremely low. However, because of the relatively low accuracy afforded by BAC arrays, many neocentromere regions included in this analysis are likely to contain regions that flank or intervene between CENP-A domains, which reduced the power of this analysis. Nevertheless, these data further support the idea that DNA sequence plays little or no role in neocentromere determination.

## Discussion

A better understanding of the structure of the human centromere is important to elucidating its function and the requirements for its formation. Human neocentromeres provide the opportunity to investigate chromatin domain structure in the absence of alpha satellite DNA and relate it directly to the underlying DNA sequence, such that complete detailed maps of entire functional centromeres can be constructed. ChIP coupled with custom-made microarrays at three different resolutions was used in this report to investigate the organization of three inner kinetochore proteins CENP-A, CENP-C, and CENP-H at human neocentromeres. This work represents the highest resolution and most detailed description to date of chromatin domains across a functional human centromere.

The genomic positions of at least seven independent neocentromeres have been determined by ChIP on BAC microarrays; however, these have relatively limited resolution because of the large size and overlaps of BACs [30-33,39]. At the neocentromere in cell line BBB, CENP-A, CENP-C, and CENP-H ChIP DNA exhibited essentially identical hybridization signals on two contiguous BACs (Figure 1), which was previously interpreted to indicate a continuous approximately 130 kb chromatin domain [32]. In contrast, at the neocentromere in cell line IMS13q, CENP-A ChIP DNA hybridized to two contiguous BACs, whereas CENP-C and CENP-H ChIP DNA hybridized to only one, which suggested that the CENP-A domain extended about 146 kb beyond an approximately 73 kb domain that contained all three proteins (Figure 1). CENP-C ChIP and BAC array analysis of a neocentric 13q21 ring chromosome also indicated a surprisingly small CENP-C domain (54 kb) [33], although the CENP-A domain was not determined in this cell line and may extend beyond the CENP-C domain, analogous to IMS13q. However, the lack of complete overlap of CENP-A with CENP-C and CENP-H may reflect less efficient immunoprecipitation of CENP-C and CENP-H associated DNA compared with CENP-A associated DNA. Thus, smaller minor domains of CENP-C and CENP-H may be difficult to detect within the larger CENP-A domains at the sensitivity afforded by BAC arrays. Therefore, in order to further investigate and accurately define the neocentromere chromatin domains in cell line BBB, PCR microarray and qRT-PCR analyses were used. In contrast to the 130 kb continuous CENP-A domain previously defined using BAC arrays, this higher resolution analysis identified two independent CENP-A chromatin domains: a major domain of about 87.8 kb and a minor domain of about 13.2 kb, separated by approximately 158 kb (Figures 2 and 3).

Co-immunoprecipitation studies of CENP-A nucleosomes from endogenous centromeres in HeLa cells showed that CENP-C and CENP-H, as well as CENP-B and more than 30 other proteins, were tightly associated with CENP-A chromatin [9-11,27]. Therefore, we took advantage of the low-copy DNA at neocentromeres to address further the association of CENP-C and CENP-H with CENP-A directly on the underlying DNA sequence. We found precise co-localization of all three CENPs at both the major and minor domains, which defined a unique inner kinetochore chromatin domain structure. Evidence suggests that CENP-H is dependent on CENP-A for localization, and that it may bridge the interaction between CENP-C and CENP-A [7,12,16,40,41]. At endogenous human centromeres, both CENP-A and CENP-C are bound to alpha satellite DNA [13-15,26,27], although clearly at the neocentromere they bind to non-alpha satellite DNA sequences. CENP-B binds to the 17 bp degenerate CENP-B box present in alpha satellite DNA, and may determine CENP-A nucleosome positioning and aid in the formation of alpha satellite derived human artificial chromosomes [42-45]. However, centromeric chromatin at neocentromeres has assembled on regions devoid of alpha satellite DNA and CENP-B [30-32], and thus it is independent of any CENP-B assembly effect.

An in-depth sequence analysis remains consistent with a possible role for L1 sequences in neocentromere formation, especially because the only 'conserved' element that could be found in common across the complete set of seven neocentromeres for which CENP ChIP has been performed is a 70 bp motif that is part of relatively young L1 elements. This element is even found in neocentromeres with no clear enrichment of L1 sequences (Figure 5 and Additional data file 1). Additional high-resolution mapping of discontinuous CENP-A domains across other neocentromeres may reduce the background of intervening regions that do not bind CENP-A and further increase the predictive power of such sequence analysis.

The PCR microarray and qRT-PCR data presented here may reflect differences in the density of chromatin that contains CENP-A, CENP-C, and CENP-H across the major and minor domains, suggesting lower density near the edges of the domains and increasing density toward the center (Figures 2 and 3). Direct analysis of two regions using an oligomer array confirmed differences in CENP-A density near the edge and middle of the major domain (Figure 4). Small regions of reduced intensity ratios for CENP-A, CENP-C, and CENP-H were observed across the major domain (Figure 2), suggesting possible further organization into subdomains ranging in size from about 1 kb up to approximately 35 kb (Figure 4a), separated by domains no larger than 4 to 5 kb with greatly reduced density of CENP-A, CENP-C, and CENP-H chromatin. The other nucleosomes in these regions presumably contain histone H3 instead of CENP-A, although this could not be assayed directly in these cells because of the background from three additional homologous chromosomal regions that were not forming a neocentromere [35]. Although we do not formally know the absolute amount of CENP-A nucleosomes in these regions and the extent to which they are interspersed with H3 nucleosomes, the PCR microarray clearly shows differences in CENP-A, CENP-C, and CENP-H density across the domain. It would be interesting to determine whether the different CENP-A density reflects populations of cells in different stages of the cell cycle in the unsynchronized cells examined, where CENP-A density would be highest in the region after mitosis [46].

It is of interest to compare these results with the previous description of the organization of chromatin domains on the mardel10 neocentromere [34] (Figure 5). One of the most important differences is that the CENP-H domain on the mardel10 was mapped (using BAC microarrays) to an approximately 900 kb domain, which was about 1 Mb from the 330 kb CENP-A domain [39], in distinct contrast to the co-localization we observed between CENP-A and CENP-H at two chromosome 13 neocentromeres (Figures 1 and 2). PCR fragment mapping of the CENP-A domains on the mardel10 neocentromere, performed at somewhat lower resolution relative to this study, showed a central large CENP-A domain of 51.8 kb flanked on both sides by three symmetrically distributed smaller domains of 10.5 kb to 29.8 kb, separated by domains of 20.5 kb to 49.5 kb of H3 containing domains (Figure 5a). Our neocentromere showed in contrast one large major CENP-A domain and a smaller minor domain (Figure 5b). Our PCR microarray did suggest putative subdomains of CENP-A across the major domain that were more consistent with the mardel 10 domains, but with much smaller intervening regions of under 5 kb that did not appear to be completely devoid of CENP-A.

Discontinuous domains of CENP-A chromatin have now been described at human and *Drosophila *centromeres [23], rice centromeres (*Oryza sativa*) [47-49], and now at two human neocentromeres [34] (the present report also). The CENP-A chromatin at the BBB neocentromere falls within intergenic regions, where the major and minor CENP-A domains did not contain any genes, while the intervening region between the CENP-A domains, as well as the flanking regions, were relatively gene rich (Figure 2). Rice centromeres (Cen3 and Cen8) have formed on approximately Mb sized regions that have a low content of centromeric repeated DNA (CentO) and active genes. However, rice centromere chromatin appears to be largely intergenic [47-49]. These data may suggest an avoidance of active chromatin by CENP-A nucleosomes [28,32]. In contrast, the mardel10 neocentromere CENP-A domain has formed directly on the *ATRNL1 *gene, although notably avoiding the promoter region [39].

Discontinuous domains of CENP-A chromatin are consistent with a higher order chromatin looping model, in which domains of CENP-A chromatin are brought together to form a surface for kinetochore assembly, with intervening non-CENP-A chromatin facing inward toward the sister chromatid cohesion domains of the centromere [23,24,34]. The domain organization observed at the BBB neocentromere may suggest a single chromatin loop closed off by the major and minor CENP-A domains, although the putative subdomains of differing CENP-A density may suggest additional chromatin loops. These data taken together suggest that functional neocentromeres may form on varied patterns of inner kinetochore chromatin, and so what they have in common with each other and with endogenous centromeres may reveal considerable insight into the requirements for centromere formation and function. Further application of the technology presented here may eventually permit construction of a complete structural map of a functional neocentromere as it relates to the underlying DNA sequence, complete with the positions of heterochromatin and sister chromatid cohesion domains.

## Materials and methods

### Antibodies

Mouse monoclonal anti-CENP-A and rabbit polyclonal anti-CENP-H were previously described [9,20,27]. Rabbit polyclonal anti CENP-C was a gift from Bill Earnshaw (Institute of Molecular and Cell Biology, Edinburgh, Scotland) [18]. Mouse and rabbit IgG were obtained from Vector Laboratories (Burlingame, CA, USA).

### Chromatin immunoprecipitations

Epstein-Barr virus transformed lymphoblast IMS13q and fibroblast BBB were grown in standard media [35]. CenpA immunoprecipitation from soluble chromatin obtained by microccocal nuclease digestion - one to five nucleosomes - was done as previously described [32].

CENP-C and CENP-H immunoprecipitations were performed in cross-linked sonicated extracts. For cell line BBB six T175 flasks (about 5 × 10^7 ^cells in each) were cross-linked in 20 ml media containing 1% formaldehyde (Fisher Scientific, Fair Lawn, NJ, USA) for 10 min at room temperature. For IMS13q, about 5 × 10^7 ^cells were cross-linked in 10 ml media containing 0.5% formaldehyde and rotated at room temperature for 10 min. In both cases, the reaction was stopped by addition of glycine to a final concentration of 0.125 mol/l for 5 min. Cells were centrifuged, washed in cold phosphate-buffered saline (4°C, 5 min, 500 *g*) and incubated in 2 ml of cold cell lysis buffer (5 mmol/l Pipes [pH 8.0], 85 mmol/l KCl, 0.5% NP40, 1 mmol/l phenylmethanesulphonylfluoride [PMSF] and protease inhibitor cocktail [Sigma, St. Louis, MO, USA]) for 10 min at 4°C. Cells were centrifuged and re-suspended in 1 ml of cold nuclei lysis buffer (50 mmol/l Tris-HCl [pH 8.1], 10 mmol/l EDTA, 1% sodium dodecyl sulfate [SDS], 0.5 mmol/l PMSF, and protease inhibitor cocktail [Sigma]), and sonicated in an ultrasonic liquid processor XL 2000 Microson™ (Misonix Incorporated, Farmigdale, NY, USA) 12 times for 10 s at power 10 in an ice/ethanol bath, to obtain a DNA ladder ranging from 500 to 1,000 bp. The lysate was centrifuged for 10 min at 12,000 *g *and 4°C. The supernatant was recovered and adjusted to 1% Triton, 2 mmol/l EDTA, 20 mmol/l Tris-HCl (pH 8.1), 150 mmol/l NaCl, and 0.1% SDS, and pre-cleared with 5 μg rabbit IgG and 2% of blocked Protein G (Amersham Biotech, Piscataway, NJ, USA) for 20 minutes at 4°C. After centrifugation (250 *g *for 5 min at 4°C), 10 μl of specific rabbit polyclonal were added to the supernatant (input chromatin) and incubated for 4 hours at 4°C. The immunocomplexes were recovered by incubation with 6% blocked Protein G for 2 hours at 4°C, and centrifugation, and were washed consecutively with low salt buffer (0.1% SDS, 1% Triton, 2 mmol/l EDTA, 20 mmol/l Tris [pH 8.1], 150 mmol/l NaCl), high salt buffer (0.1% SDS, 1% Triton, 2 mmol/l EDTA, 20 mmol/l Tris [pH 8.1], 500 mmol/l NaCl), LiCl buffer (0.25 mol/l LiCl, 1% NP40, 1% deoxycholate, 1 mmol/l EDTA, 10 mmol/l Tris [pH 8.1]) and TE (10 mmol/l Tris, 1 mmol/l EDTA). The immunocomplexes were re-suspended in 10 mmol/l Tris HCl (pH 7.5), 5 mmol/l EDTA and 0.25%SDS, and to reverse the cross-links both input chromatin and immunocomplexes were adjusted to 200 mmol/l NaCl and incubated at 60°C for 8 hours, in the presence of 150 μg of Proteinase K PCR grade (Roche Applied Science, Indianapolis, IN, USA) and 20 μg of RNAseA (Qiagen, Valencia, CA, USA). DNA was phenol chloroform treated, ethanol precipitated with 1 μg of glycogen (Roche Applied Science) and quantified using the Nanodrop ND-1000 Spectrophotometer (Nanodrop Technologies, Wilmington, DE, USA).

### PCR amplification and labeling of chromatin DNA

For BAC and PCR microarrays, the CENP-A input and immunocomplexes were amplified using ligation-mediated aminoallyl-dUTP (Sigma) PCR, as described by Alonso and coworkers [32]. For CENP-C, and CENP-H input and immunocomplexes, 25 ng of sonicated DNA were repaired with 2.4 units of Kinase, 8 units of Klenow, and 8 units of T4 polymerase (NEB, Beverly, MA, USA) for 1 hour at 37°C, and ligation-mediated PCR was carried out with 3 ng, as described by Alonso and coworkers [32]. A mass of 5 μg of amplified aminoallyl-dUTP PCR product was conjugated with approximately 30 ng of Mono-Reactive-Cy3 (input chromatin) or Mono-Reactive-Cy5 (CenpA/CenpC/CenpH ChIP DNA) Dye Pack in 100 mmol/l NaHCO_3 _(Amersham Biosciences Piscataway, NJ, USA,) for 1 hour in the dark. The specific activity of the probes ranged from 50 to 70 nucleotides per dye. For the oligo microarray, 5 μg CENP-A input and immunocomplexes obtained by ligation mediated PCR were labeled by random priming, using the Invitrogen Bioprime kit (Invitrogen, Carlsbad, CA, USA) with 20 μmol/l Cy3-dCTP or Cy5-dCTP (Amersham Biosciences). Specific activity of the probes ranged from 40 to 80 nucleotides per dye.

### 13q32 BAC microarrays

Two different BAC CHIPs were used in this study. BAC-CHIP01 contained 126 contiguous BACs spanning 14 Mb across 13q31.3 to 13q33.2 (hg17 genome coordinates chr13: 91,899,368 to 105,726,707) [50] and including all BACs from proximal RP11-165N12 (AL159152) to distal RP11-358P11 (AL139379). BAC DNA was purified, sonicated, re-suspended in 50% DMSO at approximately 200 ng/μl, and spotted, as described by Alonso and coworkers [32]. BAC-CHIP02 contained 216 BACs spanning about 25 Mb across 13q31.3-13q34 (hg17 genome coordinates chr13: 88,810,221 to 114,017,025) [50], included all BACs from RP11-114G1 (AL163533) to RP11-569D9 (AL160396). There are three gaps in the last 2.5 Mb of this contig according to the UCSC genome browser hg17: 435 kb from BACs RP11-65D24 (AL359649) to RP11-75F3 (AL136302); 122 kb from BACs RP11-230F18 (AL442125) to RP11-199F6 (BX072579); and 130 kb from BACs RP11-199F6 (BX072579) to RP11-2445B11 (AL161774). BACs for BAC-CHIP02 were obtained from the RZPD German Resource Center for Genome Research (Berlin, Germany), grown in LB/kanamycin, and purified using NucleoBond columns (Clontech, Mountain View, CA, USA). Purified BAC DNA was DOP-PCR amplified, in accordance with the method reported by Fiegler and coworkers [51]. DNA was cleaned using standard techniques and re-suspended in spotting buffer (3 × SSC (150 mmol/l NaCl, 15 mmol/l Na Citrate pH 7.0) 1.5 mol/l betaine) at 100 ng/μl. Approximately 50 pg DNA was spotted onto Nexterion slides E (Schott, Mainz, Germany), using a Stealth SMP3 Microarray Spotting Pin (Telechem International, Inc., Sunnyvale, CA, USA) and Genemachine Omnigrid 100 (Genomics Solutions Inc. Ann Arbor, MI, USA). BAC microarray information, and raw and processed data can be obtained from ArrayExpress [52], under accession number E-TABM-238.

### PCR microarray

PCR amplicons with an average length of 588 bp were designed to represent the nonrepetitive sequences at the BBB neocentromere region at an average spacing of 2 kb (hg17 genome coordinates chr13: 101,814,053 to 102,158,450 Mb) and surrounding the neocentromere with a gradual increasing density of 10 to 30 kb (hg17 genome coordinates chr13: 100,832,939 to 103,159,630 Mb) [50]. Genomic DNA was extracted from peripheral blood of a healthy donor and 150 ng were PCR amplified with 300 nmol/l primers, 2.5 mmol/l MgCl, 0.2 mmol/l dNTP, and 3.5 U Eurotaq (Euroclone, Pero, Milano, Italy), in Thermo-cycler Primus HT (MWG-Biotech, Ebersberg, Germany). The amplification cycles were one cycle 96°C for 2 min; one cycle at 94°C for 2 min; 35 cycles at 94°C for 30 s, 58°C for 30 s, and 72°C for 1 min; and one cycle at 72°C for 5 min. A 100 μl PCR reaction typically yielded 800 to 1,000 ng of product, which was purified using MultiScreen^® ^PCRμ96 filter plate and a MultiScreen™ Vacuum Manifold 96-well (Millipore, Bedford, MA, USA) and vacuum dried. PCR amplified DNA was re-dissolved overnight in 15 μl spotting buffer (3 × SSC and 1.5 mol/l betaine) at an end concentration 50 to 60 ng/μl. PCR products were spotted in triplicates at approximately 25 pg of DNA onto Nexterion E slides (Schott) using a Genemachine Omnigrid 100 robot (Genomics Solutions). PCR-amplicon microarray information, and raw and processed data can be obtained from ArrayExpress [52] under accession numbers E-TABM-245 and E-TABM-257.

### Oligo microarray

A nonrepetitive 1.6 kb region at hg17 genome coordinates chr13: 101,906,612 to 101,908,221 and a 2 kb region at hg17 genome coordinates chr13: 101,937,355 to 101,939,384 [50] within the CENP-A domain on BBB were fully covered with oligonucleotides (70 mers). Oligonucleotides were obtained from Illumina (llumina Inc. San Diego, CA, USA) and spotted in triplicates in 3 × SSC and 1.5 mol/l betaine spotting solution on Nexterion E slides (Schott) using a Genemachine Omnigrid 100 robot (Genomics Solutions). Oligo-microarray information, and raw and processed data can be obtained from ArrayExpress [52] under accession number E-TABM-294.

### Microarray hybridization

For BAC and PCR arrays, approximately 5 μg from each input and ChIP were combined with 50 μg of Human Cot and 1.75 mg of yeast tRNA, and concentrated to 4 μl with a Microcon-50 (Millipore Corporation, Bedford, MA, USA). They were mixed with 36 μl of 50°C ULTRAhyb™ (Ambion Inc. Austin, TX, USA) denatured 10 minutes at 72°C and pre-annealed for 2 hours at 37°C. The annealed DNA was added to the slide, covered with a 22 × 22 mm LifterSlip (Erie Scientific Company, Portsmouth, NH, USA), and incubated for 16 hours at 42°C in an ArrayIt hybridization chamber (Telechem International Inc., Sunnyvale, CA, USA). The slides were washed for 30 min at 45°C in 50% formamide, 2 × SSC (pH 7.0) and 0.1% Tween20; for 15 min at 60°C in 5 × SSC and 0.5% SDS; for 10 min at 45°C in 2 × SSC (pH 7.0) and 0.1% Tween20; and 10 min at room temperature in phosphate-buffered saline and 0.1%Tween20. The arrays were imaged with an Affymetrix GMS 417 laser-based slide scanner (Affymetrix Inc. Santa Clara, CA, USA) and fluorescence intensity ratios measured using Scan Array 2.0 (Perkin Elmer, Waltham, MA, USA).

The oligo array was hybridized on a GeneTac hybridization machine (Genomic Solutions MI, USA) at 42°C for 44 h with constant agitation. The slides were washed at 36°C with GeneTAC Medium Stringency Wash Buffer (40s flow and 5 min hold), followed by GeneTAC High Stringency Wash Buffer (40 s flow and 4 min hold) and GeneTAC PostWash Buffer (40 s flow and 2 min hold). Slides were dried in 50 ml falcons using a centrifuge (500 *g *for 5 min) and imaged using a GenePix 4000B (Molecular Devices, Sunnyvale, CA, USA) and analyzed using GenePix Pro 5.1.0.1 (Molecular Devices).

### Statistical analysis

For each ChIP on CHIP the triplicate or quadruplate spots on the microarray were averaged using the mean Cy-5:Cy-3 normalized intensity ratios (Lowess), and spots with a greater than 25% standard deviation (SD) from the mean were rejected. Linear intensity ratios were then transformed to log_2 _ratios, scale normalized (X-log_2 _mean of experiment/SD of the experiment) [53]. For each experiment at least three independent biologic replicates were performed and averaged. For the BAC CHIPs, for which less than 1% of the values were positives, we calculated the experimental mean and SD using all values. Positive intensity ratios were identified as those that were three or more times the SD from the experimental mean. For the PCR CHIPs, for which the number of positives was about 15%, a one-tailed distribution was used [36] to calculate the experimental mean. The total range of intensity ratio values was divided into 16 bins (which is the square root of the number of observations [257]), resulting in two distinct distributions containing a large number of low values and relatively small number of higher values. The mean of the experiment was calculated using the lower values from the larger distribution. Experimental positives were taken as values whose mean log_2 _intensity ratio was greater than or equal to the experimental mean plus three times its SD.

### Quantitative real-time PCR

Each primers set was tested on a serial DNA dilution to produce a standard curve, and similar slopes were obtained for all of them. Primer sequences, coordinates, and amplified fragment sizes can be found in Additional data file 3. Reactions were performed using SYBR Green I 10,000× at a concentration of 50× (Invitrogen). Each qRT-PRC was performed in a 10 μl volume using equal amounts of input and ChIP DNA (2 to 5 ng), 20 mmol/l Tris HCl (pH 8.4), 5 mmol/l MgCl_2_, 0.2 mmol/l dNTPs, 0.2 μmol/l of each primer, and 0.025U of Platinum Taq DNA polymerase (Invitrogen). Triplicate samples were loaded in 384-well plate, placed in an ABI PRISM 7900HT (Applied Biosystems, Foster City, CA, USA), and Taq polymerase was activated by incubation at 95°C for 2 min. The amplification cycles were 30 cycles of 95°C for 15 s, 55°C for 20 s, and 72°C for 30 s, followed by an extension at 72°C for 5 min. Increased SYBR Green fluorescence was measured using SDS software version 2.1 (Applied Biosystems). The mean for the Cycle threshold (Ct) value for each triplicate was calculated. The difference in cycle threshold (ΔCt) between Input DNA and CENP-A ChIP DNA for each primer pair was calculated. To calculate the fold enhancement, the ΔCt was converted to absolute DNA content difference, 1.93^ΔCt ^[54]. For each independent ChIP experiment, the absolute DNA content difference was normalized to the value obtained for the positive control alpha satellite DNA primer pair. Biologic replicates were obtained by averaging the normalized values.

### Sequence analysis

The two CENP-A domains in cell line BBB, their intervening sequences, and the 5' and 3' sequences were analyzed using Repeat Masker [55] with the following hg17 genome coordinates: 5' end, chr13: 101,749,991 to 101,899,990; CenpA domains, chr13: 101,899-991 to 101,998,697 and chr13: 102,149,980-102,158,450; intervening sequences, chr13: 101,998,698 to 102,149,979; and 3' end, chr13: 102,158,451 and 102,308,451 [50]. The 2.5 Mb sequences including and surrounding the neocentromere domains used to analyze the abundance of L1s correspond to the following hg18 genome coordinates: BBB, chr13: 100,732,939 to 103,259,630; 98RO16, chr13: 102,188,865 to 104,688,865; IMS13q, chr13: 95,291,790 to 97,791,790; CenpA domain, chr13: 96,434,923 to 96,648,656; CenpA/C/H domain, chr13: 96,575,483 to 96,648,656; RingA, chr13: 66,617,024 to 69,117,02469; CHOP13q, chr13: 69,591,760 to 72,091,800; mardel10, chr10: 115,867,883 to 118,367,883; D1, chr10: 116,949,436 to 116,964,165; D2, chr10: 116,984,701 to 117,011,753; D3, chr10: 117,049,201 to 117,063,568; D4, chr10: 117,085,770 to 117,137,563; D5, chr10: 117,163,769 to 117,174,614; D6, chr10: 117,196,394 to 117,226,247; D7, chr10: 117,275,839 to 117,286,330; and CenpH domain, chr10: 115,058,210 to 115,833,955 [50].

### Oligomeric sequence analysis

Using a progressive search for the longest shared oligomer, we identified a 70 mer motif ATACCCAGTAATGGGATGGCTGGGTCAAATGGTATTTCTAGTTCTAGATCCCTGAGGAATCGCCACACTG as the longest perfectly conserved motif between the seven neocentromere domains, and determined the distribution of this 70 mer along chromosomes 13 and 15. We found all 9 mers present in the seven neocentromere binding sites, as well as on chromosomes 13 and 10, and determined their frequencies in both the neocentromeres and the entire chromosome. Complementary 9 mers were counted as the same oligomer. For these analyses, we have combined the discontinuous CENP-A domains from the neocentromeres from cell line BBB and the mardel10 chromosome into one sequence. We extracted the list of oligomers that are present in at least one copy in all seven neocentromere binding sites ('shared' 9 mers). Finally, using six neocentromere binding sites (BBB, IMS13q, 98RO16, CHOP13q, RingA, and mardel10) we tested whether any of these shared 9 mers are over-represented compared with the chromosomal average. Because the RingA site reports only the location of CENP-C, and the actual binding site of CENP-A is likely to be larger, we included 50 kb upstream and downstream of the site in the analysis. Additionally, we excluded the neo20 neocentromere from the analysis, because it is mapped considerably less accurately than the others (and it is likely to include a large amount of sequence that is not part of the true Cenp-A binding site). To test whether a 9 mer is significantly more abundant in a neocentromere than expected based on its abundance on the entire chromosome, we used the test described by Trindade and coworkers [38], with significance level set to *P *< 0.05.

## Additional data files

The following additional data are available with the online version of this paper. Additional data file 1 shows a sliding window analysis of LINE1 density at neocentromeres. Additional data file 2 shows an analysis of the A+T content at neocentromeres. Additional data file 3 provides 34 qRT-PCR primer pairs.

## Supplementary Material

Additional File 1 Sliding window analysis of LINE1 density at neocentromeres. Click here for file

Additional File 2 Analysis of the A+T content at neocentromeres. Click here for file

Additional File 3 Thirty-four qRT-PCR primer pairs (Figure 3). Click here for file
