# Supplementary figures and images for: Co-localization of CENP-C and CENP-H to discontinuous domains of CENP-A chromatin at human neocentromeres
Source: Genome Biol. 2007 Jul 25;8(7):R148. doi: 10.1186/gb-2007-8-7-r148 (PMC2323242; doi:10.1186/gb-2007-8-7-r148)

## Additional data file Figure 1

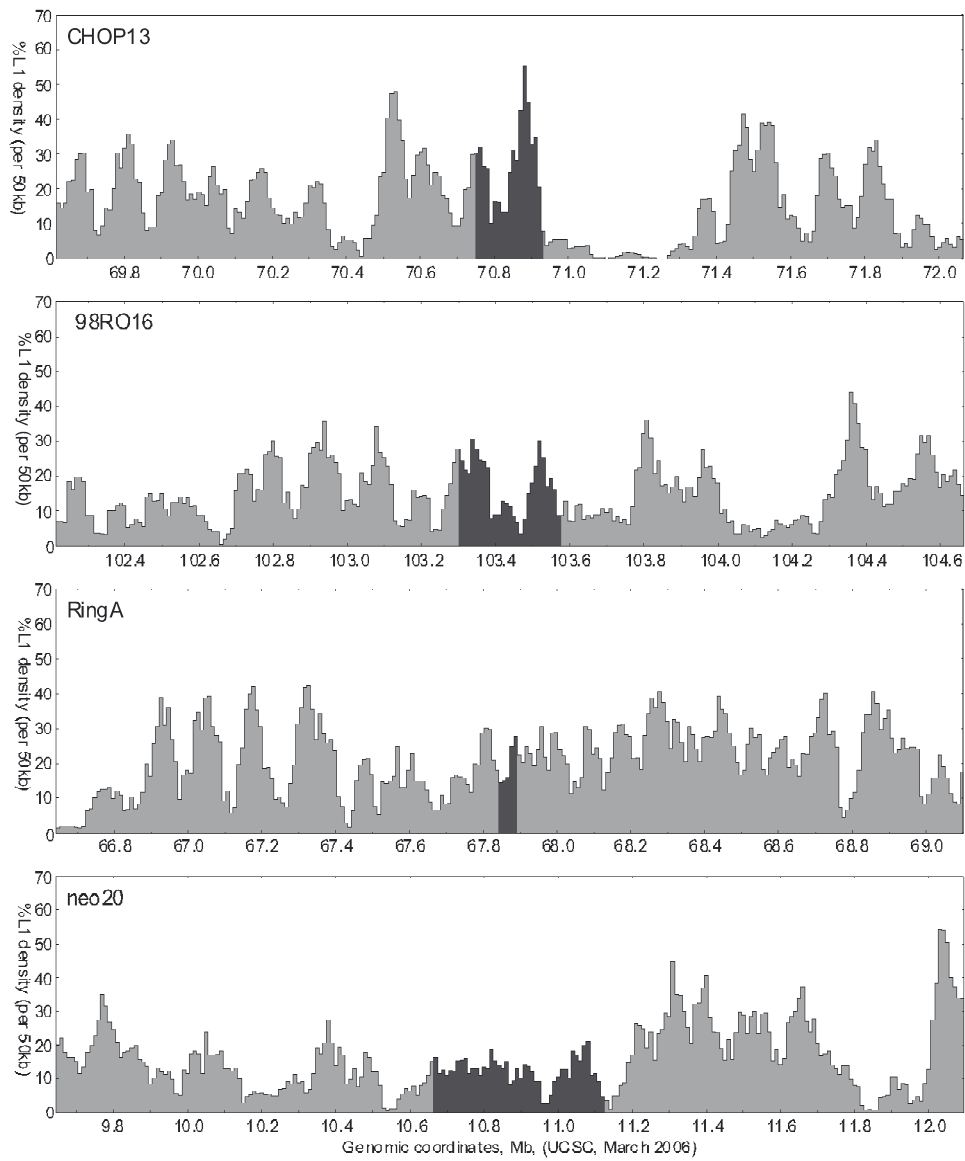

Supplement: Additional File 1 — Sliding window analysis of LINE1 density at neocentromeres. [file gb-2007-8-7-r148-S1.pdf]
