# Supplementary material for: Co-localization of CENP-C and CENP-H to discontinuous domains of CENP-A chromatin at human neocentromeres
Source: Genome Biol. 2007 Jul 25;8(7):R148. doi: 10.1186/gb-2007-8-7-r148 (PMC2323242; doi:10.1186/gb-2007-8-7-r148)
Supplement: Additional File 2 — Analysis of the A+T content at neocentromeres. [file gb-2007-8-7-r148-S2.pdf]

Additional data file Figure 2

A

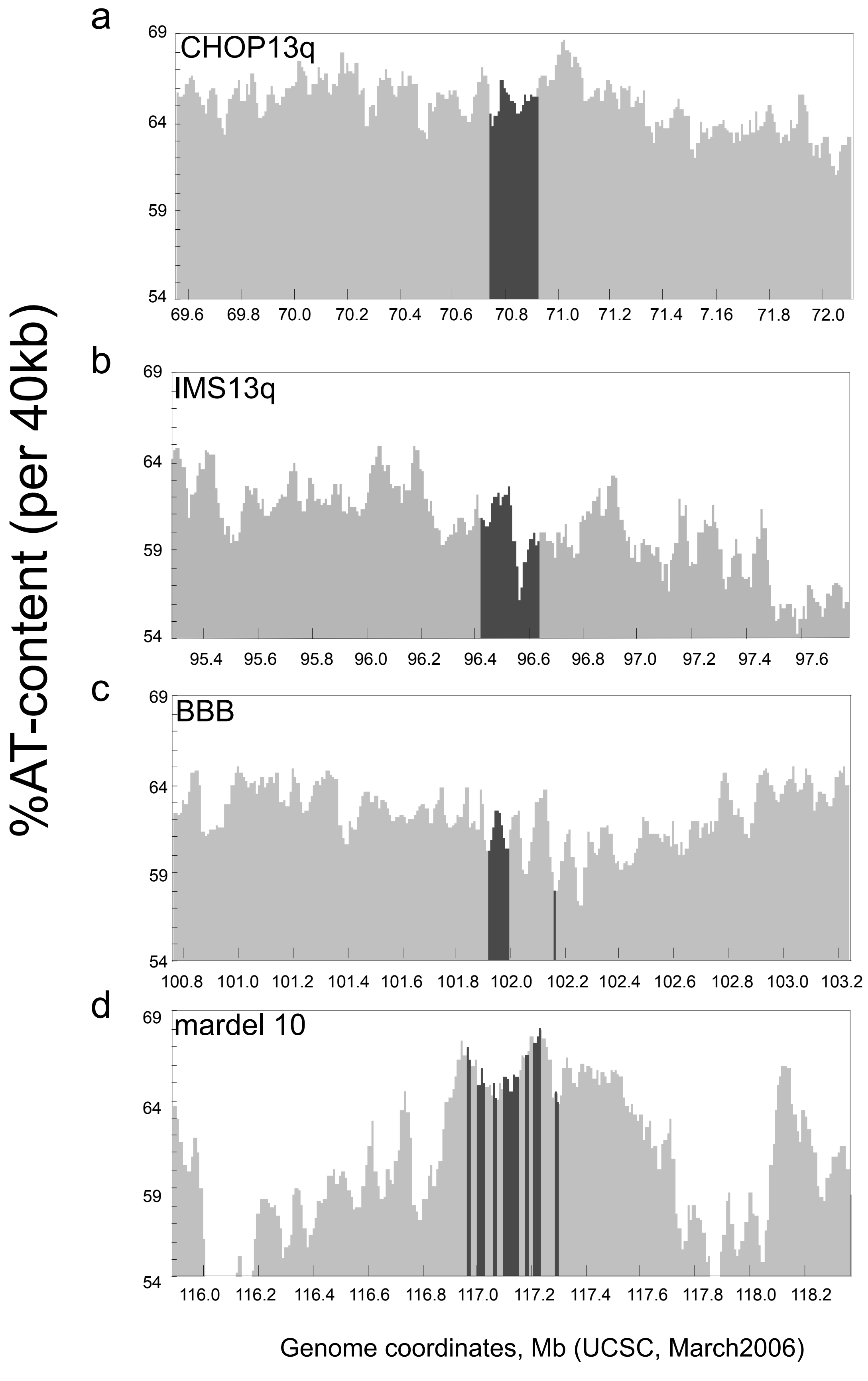

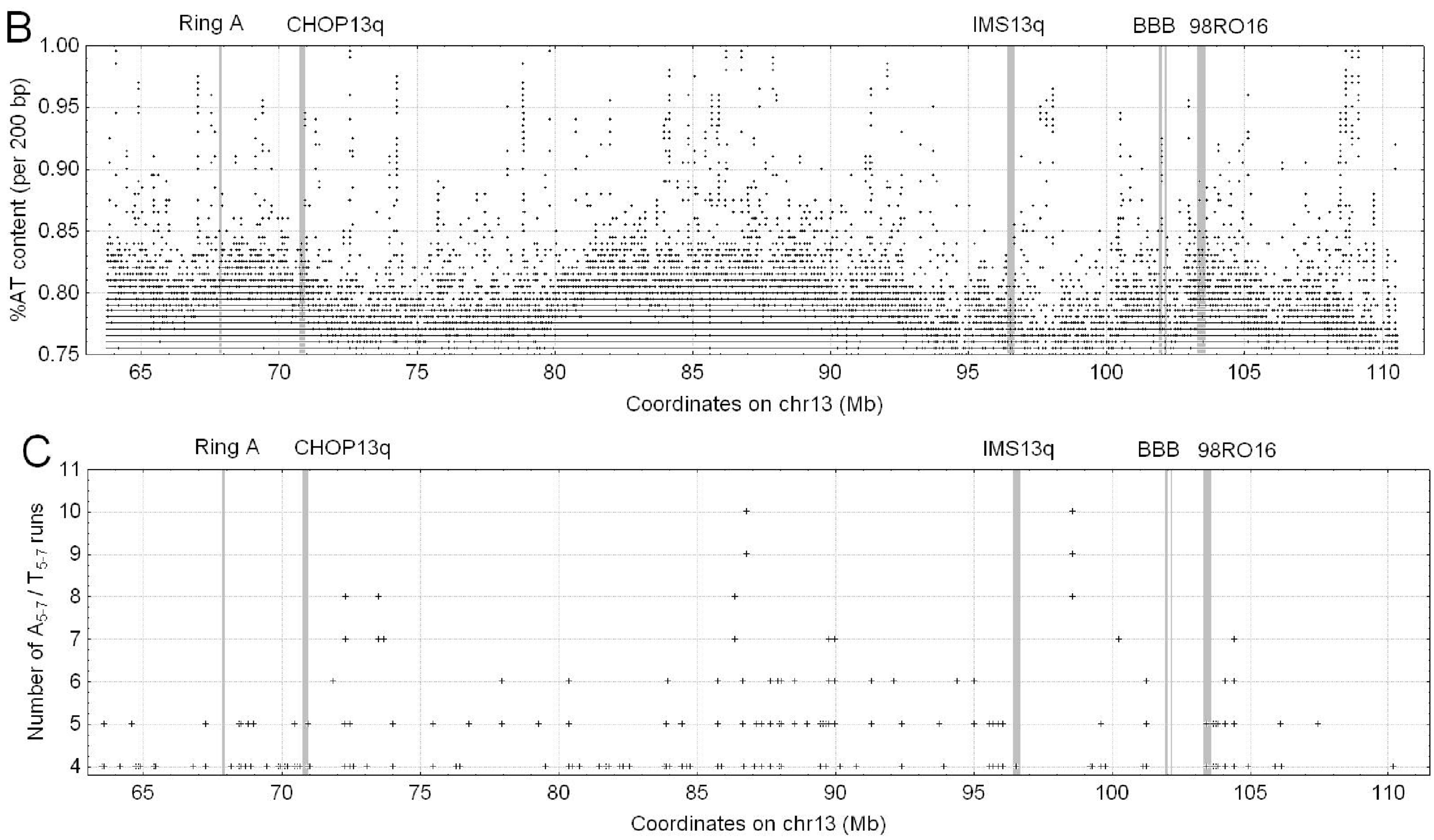

Additional data file Figure 2. Analysis of the A+T content at neocentromeres. A. The AT-content of 40 kb windows shifted every 10kb are shown across 2.5MB regions centered on human neocentromeres which have been localized by ChIP analysis. The UCSC genome coordinates hg18 (March 2006) for each region are shown on the x axis, AT-content as percentage of bp per each 40 kb window are shown on the y axis. CENP-A domains for each neocentromere is indicated by the shaded regions. a. CHOP13q. b. IMS13q. c. BBB. d. mardel10. B. The AT-content of 200bp windows shifted every 50bp across the 50Mb chr13 region containing the 5 described neocentromeres. The UCSC genome coordinates hg 18 (March 2006) are shown on the x axis, AT-content as percentage of bp per each 200bp window are shown on the y axis. Shaded regions indicate the position of neocentromeres. C. Sequences approximating the S. cerevisiae functional centromere CDEII sequences (≥four runs of A5-7/T5-7 in 90 bp of ≥90% A or T) [37] (y axis) were located for the 50Mb chr13 region containing the 5 described neocentromeres. The UCSC genome coordinates hg 18 (March 2006) are shown on the x axis.
