# Supplementary material for: Co-localization of CENP-C and CENP-H to discontinuous domains of CENP-A chromatin at human neocentromeres
Source: Genome Biol. 2007 Jul 25;8(7):R148. doi: 10.1186/gb-2007-8-7-r148 (PMC2323242; doi:10.1186/gb-2007-8-7-r148)
Supplement: Additional File 3 — Thirty-four qRT-PCR primer pairs (Figure 3). [file gb-2007-8-7-r148-S3.doc]

**Additional data file** Table 1: 34 qRT-PCR primer pairs (Figure 3).

| **qRT-PCR name (a)** | Size **(bp)** | **Forward** | **Reverse** | **UCSC HG17**  **genome coordinates** |
| --- | --- | --- | --- | --- |
| A | 232 | TGGTCTAAAAAGGTTTGTGAGG | CCCCTAGGAGACACCAACAT | 101854350-101854582 |
| B | 184 | GCAGATCTCTCAGCAGAAACC | TGGTGGCTTGATAGTGGTGA | 101895141-101895325 |
| (1) | 248 | CCCTATTCATTTCTGGGAAGC | ATGGAGCTCTGTCCTCATGC | 101900060-101900307 |
| (2) | 151 | GTGACAGCTCTTTGGGGAAA | GGCTAATCTTCATGGCCAAA | 101901806-101901956 |
| C | 178 | TTCAGAGACCCAGTCAATCG | TTGAACTTCCATTTATGTTCCTCTC | 101905979-101906157 |
| (3) | 214 | TCTCTGATTTTGCAACCTG | GGGTGTGGATTTCGTTATGG | 101906962-101907175 |
| D | 223 | ATGCTACCACTGTGCCAGGT | TGGGAAGAGGATGGCAATTA | 101912608-101912831 |
| (11) | 247 | AATCTGTGCTGGTGCAAACA | CTCCTCCTCACAGTAGCCAGTT | 101915574-101915821 |
| E | 205 | CACAAAGTGGAGGACAAGCA | ATCCCTGCCCATTTTTGAA | 101921287-101921492 |
| F | 157 | TAAAATATCAGAATGACACCAGAGAAG | TTGTTATAGTAGAACTGCCGATCA | 101939113-101939270 |
| G | 268 | TGTGCAATATGACAACCAGGA | TAGTGGTTGCCAGGGCATAG | 101950940-101951208 |
| (27) | 190 | TGTGGCACAATATGGCTGTT | AGCAACCCTGAAGATGGATG | 101954355-101954545 |
| H | 197 | TCCTCCACAAAACATGGGTAG | GATGAATCAGATTAACCAGCTCCT | 101968256-101968453 |
| (35-1) | 173 | GAGATGTAACCATCACCACTTCC | CATTTCTTTGGGTTGAATCTTTC | 101980045-101980217 |
| (35-2) | 203 | GAATGAAATCCCACACAACAAA | TCGTTTTCCTCTCCGTCCTA | 101980220-101980423 |
| (38) | 143 | CTGCAGGTGGATGAAGGAAT | ATATCAAGCAGGGCAGCAAT | 101985349-101985492 |
| I | 283 | TGACAGCAGATTTCTCAGCAG | AGGTCCTGTGTGCTTTAGGC | 101993191-101993474 |
| J | 163 | AGTGGTCACAGGGAAAGCTG | GCTCTGACCTCTTCGATGCT | 102000980-102001143 |
| K | 218 | TACAACAGCATGCCAAGGAG | GTCTGGGAAGCACCAGGATA | 102002878-102003096 |
| L | 173 | GGAGACGGCAATTGAGAAGA | TGCCGTTGATGTTTATTGGA | 102006552-102006725 |
| M | 193 | CAGAGACAAGTATTTGGAAAAGG | CGCAAGAAAGACCATTGGAT | 102013402-102013595 |
| N | 255 | TCCATGCCTTGCCTAATAGC | CGTGTCACCGTTGTACATGA | 102020369-102020624 |
| O | 199 | TCTTCAGGGAGTGGTCTGCT | TTAAATCCTAGAAAATGCGTCTTG | 102030972-102031171 |
| P | 294 | CAGGCATGAGCCTATCCAGT | AGCTTCTGCTTTTGGGTTGA | 102037124-102037418 |
| Q | 204 | TTCCGGAATTGTTTCCTGTG | AAACCGTCGTATGTTTTCAGG | 102048161-102048365 |
| R | 197 | TCGTGTTCTTCTGAGGTGTCA | CCCATACCAAAAAGGCAAAT | 102093587-102093784 |
| (90) | 232 | TGTACCCACCCTCGGTACTC | GTTGCAGAGATGCAGAATGC | 102145930-102146161 |
| (91) | 188 | ATGGGATGCAAAGACTGAGG | TTTCCATGTTCGCAAGTCTG | 102150236-102150423 |
| (92) | 237 | CCTGTTCCTTGGAAGCTGAG | CAGCTTCCTTGAATGGCTTC | 102151585-102151821 |
| S | 159 | TCAAGGTGCTGCCACAGTTA | CATCTCCTGCTTCAGGGTTT | 102153712-102153870 |
| T | 190 | CTGTCAATAGACTAGTAATGTCACCTG | TCCTTCTCACTGCCCTTGAT | 102155504-102155693 |
| (93) | 246 | ACACAGGGATTTCTCCCAAA | TCAACACATTCTGGGACAGG | 102158077-102158322 |
| U | 225 | AAATTCATGCAGATCAGTTTCTTT | AACTGGAAGCAAATAGGAGAAA | 102158924-102159148 |
| V | 170 | AAATGTTAGAGTTGACTGGGTAGTTT | TTGGCTATGACTTTATACTTGTTTCTC | 102165233-102165402 |
| Alpha sat  ConA/ConB | 125 | CTAGACAGAAGAATTCTCAG | CTGAAATCTCCACTTGC | chr1:  121096102-121096226 |

a) numbers in parentheses identify qRT-PCR fragment wholly contained within the PCR-microarray fragment with the same number. Letters identify all other qRT-PCR primers (Figure 3).
